# Applying compressed sensing to genome-wide association studies

**DOI:** 10.1186/2047-217X-3-10

**Published:** 2014-06-16

**Authors:** Shashaank Vattikuti, James J Lee, Christopher C Chang, Stephen D H Hsu, Carson C Chow

**Affiliations:** 1 Mathematical Biology Section, Laboratory of Biological Modeling, National Institute of Diabetes and Digestive and Kidney Diseases, National Institutes of Health, South Drive, Bethesda, MD 20814, USA; 2 Department of Psychology, University of Minnesota Twin Cities, 75 East River Parkway, Minneapolis, MN 55455, USA; 3 BGI Hong Kong, 16 Dai Fu Street, Tai Po Industrial Estate, Tai Po, Hong Kong; 4 Department of Physics and Office of the Vice President for Research and Graduate Studies, Michigan State University, 426 Auditorium Road, East Lansing, MI 48824, USA; 5 Cognitive Genomics Lab, BGI Shenzhen, Yantian District, Shenzhen, China

**Keywords:** GWAS, Genomic selection, Compressed sensing, Lasso, Underdetermined system, Sparsity, Phase transition

## Abstract

**Background:**

The aim of a genome-wide association study (GWAS) is to isolate DNA markers for variants affecting phenotypes of interest. This is constrained by the fact that the number of markers often far exceeds the number of samples. Compressed sensing (CS) is a body of theory regarding signal recovery when the number of predictor variables (i.e., genotyped markers) exceeds the sample size. Its applicability to GWAS has not been investigated.

**Results:**

Using CS theory, we show that all markers with nonzero coefficients can be identified (selected) using an efficient algorithm, provided that they are sufficiently few in number (sparse) relative to sample size. For heritability equal to one (*h*^*2*^ = 1), there is a sharp phase transition from poor performance to complete selection as the sample size is increased. For heritability below one, complete selection still occurs, but the transition is smoothed. We find for *h*^*2*^ ∼ 0.5 that a sample size of approximately thirty times the number of markers with nonzero coefficients is sufficient for full selection. This boundary is only weakly dependent on the number of genotyped markers.

**Conclusion:**

Practical measures of signal recovery are robust to linkage disequilibrium between a true causal variant and markers residing in the same genomic region. Given a limited sample size, it is possible to discover a phase transition by increasing the penalization; in this case a subset of the support may be recovered. Applying this approach to the GWAS analysis of height, we show that 70-100% of the selected markers are strongly correlated with height-associated markers identified by the GIANT Consortium.

## Background

The search for genetic variants associated with a given phenotype in a genome-wide association study (GWAS) is a classic example of what has been called a *p* ≫ *n* problem, where *n* is the sample size (number of subjects) and *p* is the number of predictor variables (genotyped markers) [1]. Estimating the partial regression coefficients of the predictor variables by ordinary least squares (OLS) requires that the sample size exceed the number of coefficients, which in the GWAS context, may be of order 10^5^ or even 10^6^. The difficulty of assembling such large samples has been one obstacle hindering the simultaneous estimation of all regression coefficients advocated by some authors [2-4].

The typical procedure in GWAS is to estimate each coefficient by OLS independently and retain those meeting a strict threshold; this approach is sometimes called *marginal regression* (MR) [5]. Although the implementation of MR in GWAS has led to an avalanche of discoveries [6], it is uncertain whether it will be optimal as datasets continue to increase in size. Many genetic markers associated with a trait are likely to be missed because they do not pass the chosen significance threshold [7].

Unlike MR, which directly estimates whether each coefficient is nonzero, an *L*_1_-penalization algorithm, such as the lasso, effectively translates the estimates toward the origin where many are truncated out of the model [8]. If the number of variants associated with a typical complex trait is indeed far fewer than the total number of polymorphic sites [9-11], then it is reasonable to believe that *L*_1_ penalization will at least be competitive with MR. Methods relying on the assumption of *sparsity* (few nonzero coefficients relative to sample size) have in fact been adopted by workers in the field of genomic selection (GS), which uses genetic information to guide the artificial selection of livestock and crops [12-15]. Note that the aim of GS (phenotypic prediction) is somewhat distinct from that of GWAS (the identification of markers tagging causal variants). The lasso is one of the methods studied by GS investigators [16,17], although Bayesian methods that regularize the coefficients with strong priors tend to be favored [18,19].

In this paper we show that theoretical results from the field of *compressed sensing* (CS) supply a rigorous quantitative framework for the application of regularization methods to GWAS. In particular, CS theory provides a mathematical justification for the use of *L*_1_-penalized regression to recover sparse vectors of coefficients and highlights the difference between *selection* of the markers with nonzero coefficients and the *fitting* of the precise coefficient values. CS theory also addresses the robustness of *L*_1_ algorithms to the distribution of nonzero coefficient magnitudes.

Besides supplying a rule of thumb for the sample size sufficing to select the markers with true nonzero coefficients, CS gives an independent quantitative criterion for determining whether a given dataset has, in fact, attained that sample size. Whereas biological assumptions regarding the number of nonzeros do enter into the rule of thumb about sample size, these assumptions need not hold for the use of *L*_1_ penalization to be justified; this is because the returned results themselves inform the investigator whether the assumptions are met.

We emphasize that CS is not a method *per se,* but may be considered a general theory of regression that takes into account model complexity (sparsity). The theory is still valid in the classical regression domain of *n* > *p* but establishes conditions for when full recovery of nonzero coefficients is still possible when *n* < *p*[20-22]. Our work therefore should not be directly compared to recent literature proposing and evaluating GS methods [18,19]. Rather, our goal is to elucidate properties of well-known methods, already in use by GWAS and GS researchers, whose mathematical attributes and empirical prospects may be insufficiently appreciated.

Using more than 12,000 subjects from the Atherosclerosis Risk in Communities Study (ARIC) European American and Gene-Environment Association Studies (GENEVA) cohorts and nearly 700,000 single-nucleotide polymorphisms (SNPs), we show that the matrix of genotypes acquired in GWAS obeys properties suitable for the application of CS theory. In particular, a given sample size determines the maximum number of nonzeros that will be fully selected using an *L*_1_-penalization regression algorithm. If the sample size is too small, then the complete set of nonzeros will not be selected. The transition between poor and complete selection is sharp in the noiseless case (narrow-sense heritability equal to one). It is smoothed in the presence of noise (heritability less than one), but still fully detectable. Consistent with CS theory, we find in cases with realistic residual noise that the minimal sample size for full recovery is primarily determined by the number of nonzeros, depends very weakly on the number of genotyped markers [22-24], and is robust to the distribution of coefficient magnitudes [25].

### Theory of compressed sensing

The linear model of quantitative genetics is

(1) y=Ax+e

Where **y** ∈ ℝ^*n*^ is the vector of phenotypes, **A** ∈ ℝ^*n*x*p*^ is the matrix of standardized genotypes, **x** ∈ ℝ^*p*^ is the vector of partial regression coefficients, and **e** ∈ ℝ^*n*^ is the vector of residuals. In the CS literature, **A** is often called the *sensing* or *measurement* matrix. Standardizing **A** does not affect the results and makes it simpler to utilize CS theory. We suppose that **x** contains *s* nonzero coefficients (“nonzeros”) whose indices we wish to know.

The phase transition to complete selection is best quantified with two ratios (*ρ*, *δ*), where *ρ* = *s*/*n* is a measure of the sparsity of nonzeros with respect to the sample size and *δ* = *n*/*p* is a measure of the undersampling. If we plot *δ* on the abscissa ( *x*-axis) and *ρ* on the ordinate (*y*-axis), we have a *phase plane* on the square (0, 1) × (0, 1), where each point represents a possible GWAS situation (sample size, number of genotyped markers, number of true nonzeros). The performance of any given method can be assessed by evaluating a measure of recovery quality at each point of the plane. For an arbitrary *p*-vector **x**, we use the following notation for the *L*_1_ and *L*_2_ norms:

$${∥\;x\;∥}_{L_{1}}=\sum_{i=1}^{p}\left|\;x_{i\;}\right|\;\mathrm{and}\;{∥\;x\;∥}_{L_{2}}=\;\sqrt{\sum_{i=1}^{p}x_{i}^{2}}$$

Our results rely on two lines of research in the field of CS, which we summarize as two propositions.

**Proposition 1 **[20,24,26,27]* Suppose that the entries of the sensing matrix ****A *** *are drawn from independent normal distributions and ****e *** *is the zero vector (noiseless case). Then the ρ* − *δ plane is partitioned by a curve *$\rho =\rho_{L_{1}}\left(\delta \right)$* into two phases. Below the curve the solution of *$\min_{\hat{x}}\;{∥\;\hat{x}\;∥}_{L_{1}}$* subject to *$A\;\hat{x}=y$* leads to *$\hat{x}=x$* with probability converging to one as n*, *p*, *s* → ∞ *in such a way that ρ and δ remain constant. Above the curve *$\hat{x}\neq x$* with similarly high probability.*

**Figure 1:**
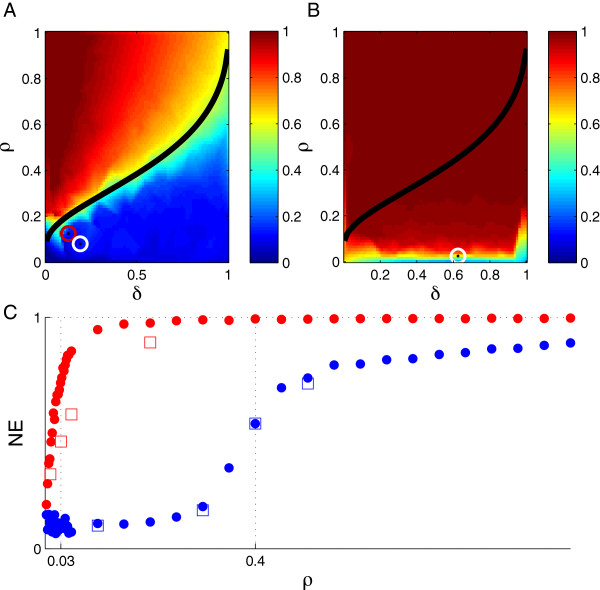
**Error in the *ρ*** **−** ***δ *plane for a measurement matrix of random genomic SNPs (**$\rho =\frac{s}{n}$** and **$\delta =\frac{n}{p}$**).** (A) Color corresponds to the normalized error (*NE*) of the coefficients $\frac{{∥x-\hat{x}∥}_{L2}}{{∥x∥}_{L2}}$. The black curve is the expected phase boundary between poor and good recovery from [26]. The number of SNPs, *p*, was fixed at 8,027. The heritability was set to one (noiseless case). The circles correspond to the points (*ρ* = 0.08, *δ* = 0.19) (white) and (*ρ* = 0.125, *δ* = 0.125) (red) discussed in Measures of selection. **(B)** Same as panel **(A)**, except that the heritability was set to 0.5 (noisy case). The white circle corresponds to the point (*ρ* = 0.025, *δ* = 0.625) discussed in Measures of selection. **(C)***NE* versus *ρ* for fixed *n* = 4,000 and *p* = 8,027 (blue corresponds to *h*^2^ = 1, red to *h*^2^ = 0.5). The square markers indicate recovery quality evaluated at a few data points using the lasso algorithm with 10-fold cross-validation written by MATLAB.

The function $\rho_{L_{1}}\left(\delta \right)$ can be analytically calculated [26]. Although Figure 1A presents some of our empirical results, which we will discuss below, it can be taken as an illustration of the meaning of Proposition 1. The color scale represents the goodness of recovery, and the black curve is the graph of $\rho_{L_{1}}\left(\delta \right)$. It can be seen that increasing the sample size relative to *s* (decreasing *ρ*) leads to a sharp transition from poor to good recovery for *δ* < < 1 (i.e. *n* < < *p*). In other words, despite the fact that solving for **x** in **Ax** = **y** is strictly speaking underdetermined given *n* < *p*, minimizing $||\hat{x}|{|}_{L_{1}}$ subject to the system of equations still yields recovery of **x** with high probability if *n* is sufficiently large relative to *s*.

Most phenotypes do not have a heritability of one and are therefore, not noiseless, but CS theory shows that selection is still possible in this situation. Before stating the relevant CS result, we need to define two quantities characterizing the genotype matrix **A.**

**Definition 1 **[22]* The matrix ****A *** *satisfies isotropy if the expectation value of ****A ****’ ****A *** *is equal to the identity matrix.*

In the context of GWAS, a matrix of gene counts is isotropic if all markers are in linkage equilibrium (LE).

**Definition 2 **[22]* The coherence of the matrix ****A *** *is the smallest number γ such that, for each row ***a***of the matrix,*

$$\max_{1\leq t\leq p}|a_{t}{|}^{2}\leq \gamma$$

Thus, a matrix of genotypes is reasonably *incoherent* if the magnitudes of the matrix elements do not differ greatly from each other. In the GWAS context, **A** will be reasonably incoherent if all markers with very low minor allele frequency (MAF) are pruned, since **A** is standardized and the standard deviation scales with MAF.

We can now state

**Proposition 2 **[22]* Suppose that the sensing matrix ****A *** *is isotropic with coherence γ. If n* > *C γ s* log *p for a constant C then the solution of the problem*

$$\min_{\hat{x}}\left[{∥y\;-A\hat{x}∥}_{L_{2}}^{2}\;+\;\lambda {{∥\;\hat{x}\;∥}_{L}}_{{}_{1}}\right]$$

*with a suitable choice of ** obeys*

$${∥\hat{x}\;-\;x∥}_{L_{2}}^{2}\leq \frac{\sigma_{E}^{2}}{n}s\;\mathrm{poly}\log\left(p\right)$$

*where *$\sigma_{E}^{2}$* is the variance of the residuals in ****e****.*

Two features of Proposition 2 are worth noting. First, no strong restrictions on **x** are required. Second, the critical threshold value of *n* depends linearly on *s*, but only logarithmically on *p*. For *n* larger than the critical value, the deviations of the estimated coefficients from the true values will follow the expected OLS scaling of $1/\sqrt{n}$.

These results are more powerful than they might seem from the restrictive hypotheses required for brief formulations. For example, it has been shown that a curve similar to that in Proposition 1 also demarcates a phase transition in the case of **e** ≠ 0 — although, as might be expected from a comparison of Propositions 1 and 2, with large residual noise the transition is to a regime of gradual improvement with *n* rather than to instantaneous recovery [24,28]. A remarkable feature of this gradual improvement, however, should be noted. Proposition 2 states that the scaling of the total fitting error in the favorable regime is within a polylogarithmic factor of what would have been achieved if the identities of the *s* nonzeros had been revealed in advance by an oracle. This result implies that perfect selection of nonzeros can occur before the magnitudes of the coefficients are well fit. Even if the residual noise is substantial enough to prevent the sharp transition from large to negligible fitting error evident in Figure 1A, the total magnitude of the error in the favorable phase is little larger than what would be expected given perfect selection of the nonzeros.

Recent work has also generalized the sensing matrix, **A**, in Proposition 1 to several non-normal distributions (although not to genotype matrices *per se*) [27,29]. Furthermore, the form of Proposition 2 also holds under a weaker form of isotropy that allows the expectation of **A’A** to differ from the identity matrix by a small quantity (see [22] for the specification of the matrix norm). The latter generalization is promising because the covariance matrix in GWAS deviates toward block-diagonality as a result of linkage disequilibrium (LD) among spatially proximate variants.

Whereas the penalization parameter λ in Proposition 2 is often determined empirically through cross-validation, CS places a theoretical lower bound on its value that is based on the magnitude of the noise [22] (referred here as *λ*_*min*_ or *λ*). A special feature of the GWAS context is that an estimate of the residual variance can be obtained from the genomic-relatedness method [7,30-32], thereby enabling the substitution of a theoretical noise-dependent bound for empirical cross-validation. Such noise-dependent bounds appear in other selection theories, including MR, and thus are not specific to CS [5,33]. As noted by [33], such bounds tend to be conservative. Here, we show that the CS noise-dependent bound demonstrates good selection properties. A data-specific method, such as cross-validation may exhibit slightly better properties, but is computationally more expensive.

Given this body of CS theory, a number of questions regarding the use of *L*_1_-penalized regression in GWAS naturally arise:

1. Does the matrix of genotypes **A** in the GWAS setting fall into the class of matrices exhibiting the CS phase transition across the curve $\rho_{L_{1}}\left(\delta \right),$ as described by Proposition 1?

2. Since large residual noise is typical, we must also ask: is **A** sufficiently isotropic and incoherent to make the regime of good performance described by Proposition 2 practically attainable? Since log *p* slowly varies over the relevant range of *p* we can absorb *γ* and log *p* into the constant factor and phrase the question more provocatively: given that *n* > *Cs* is required for good recovery, what is *C*?

3. In practice, a measure of recovery relying on the unknown **x**, such as a function of ${∥\hat{x}\;-\;x∥}_{L_{2}}$, cannot be used. Is there a measure of recovery, then, that depends solely on observables?

The aim of the present work is to answer these three questions.

## Data description

All participants gave informed consent. All studies were approved by their appropriate Research Ethics Committees.

We used the ARIC and GENEVA European American cohort. The datasets were obtained from dbGaP through dbGaP accession numbers [ARIC:phs000090] and [GENEVA:phs000091] [34]. The ARIC population consists of a large sample of unrelated individuals and some families. The population was recruited in 1987 from four centers across the United States: Forsyth County, North Carolina; Jackson, Mississippi; Minneapolis, Minnesota; and Washington County, Maryland.

The ARIC subjects were genotyped with the Affymetrix Human SNP Array 6.0. We selected biallelic autosomal markers based on a Hardy-Weinberg equilibrium tolerance of *P* < 10^− 3^. Preprocessing was performed with PLINK 2 [35,36].

The datasets were merged to create a SNP genotype matrix (**A**) consisting of 12,464 subjects and 693,385 SNPs. SNPs were coded by their minor allele, resulting in values of 0, 1, or 2. Each column of **A** was standardized to have zero mean and unit variance. Missing genotypes were replaced with the mean (i.e., zero) after standardization. We compared results for the phase transition for a limited number of cases when the missing genotypes were imputed based on sampling from a Binomial distribution and the respective minor allele frequency. We found no difference between the imputation methods for our datasets.

We simulated phenotypes according to Equation 1, rescaling each term to leave the phenotypic variance equal to unity and the variance of the breeding values in **Ax** to match the target narrow-sense heritability *h*^2^, which is the proportion of phenotypic variance due to additive genetic factors. For standardized phenotypes, *h*^2^ is equivalent to the additive genetic variance, which is defined to equal one in the noiseless case. We chose *h*^2^ = 0.5 to represent the noisy case because many human traits show a SNP-based heritability close to this value [7,30,37].

The magnitudes of the *s* nonzeros in **x** were drawn from either the set {−1,  1} or hyperexponential distributions. We defined two hyperexponential distributions (Hyperexponential 1 and 2) and each was generated by summing two exponentials with the same amplitude, but different decay constants. The pair of decay constants for Hyperexponential 1 were 0.05*s* and *p*, and that of Hyperexponential 2 were 0.2*s* and *p*. The coefficients were then truncated to keep only the top *s* nonzero coefficients, the rest were made zero, and 50% of the nonzeros had negative signs. The hyperexponential form was motivated by [38], but the decay constants were arbitrarily chosen. For all coefficient ensembles, the nonzeros were randomly distributed among the SNPs. When examining the dependence of an outcome on *n, p,* and *s* the set *p* was either chosen randomly across the genome without replacement or restricted to all chromosome 22 SNPs, and *n* and *s* were randomly sampled without replacement. A single set of SNPs was used for all analyses of the genomic random *p* set.

We also considered a real phenotype (height) rather than a simulated one, using 12,454 subjects with measurements of height adjusted for sex. We examined different values of *n* and fixed *p* by always using all markers in our dataset. A called nonzero was counted as a true positive in the numerator of our “adjusted positive predictive value” (to be defined later) if the marker was a member of a proxy set based on height-associated SNPs discovered by the GIANT Consortium [39]. The set was generated using the BROAD SNAP database [40]. We based our proxy criterion on basepair distance rather than LD, as we found the correlations between SNPs in our dataset to be larger in magnitude than those recorded in the SNAP database. We generated a proxy list based on a maximum basepair distance of 500 kb, which was the maximum distance that could be queried.

## Analysis

### Phase transition to complete selection

We first studied the case of independent markers to gain insight into the more realistic case of LD among spatially proximate markers [17,41]. In the noiseless case (**e** = **0**), it has been proven that there is a universal phase transition boundary between poor and complete selection in the *ρ* − *δ* plane (Proposition 1) [20,24,26,27]. The existence of this boundary is largely independent of the explicit values of *s*, *n*, and *p* for a large class of sensing matrices, including sensing matrices generated by the multivariate normal distribution. However, the transition boundary does depend, on certain properties of the distribution describing the coefficients. For example, the boundary can depend critically on whether the coefficients are all positive or can have either sign, although the particular form of the distribution within either of these two broad classes is less important. Genetic applications typically have real-valued coefficients, which are in the same class (i.e., in terms of phase transition properties) as coefficients drawn from the set {−1,  1} [25,42], which we used in the majority of our simulations. We also studied selection performance when the coefficients are hyperexponentially distributed (see Data Description).

The phase transition can be explored using multiple measures of selection quality. Figure 1A shows the normalized error (*NE*) (Equation 5) of the coefficient estimates returned by the *L*_1_-penalized regression algorithm in our study of a simulated phenotype and a random selection of SNPs ascertained in a real GWAS for the noiseless case. The boundary between poor and good performance, as evidenced by this measure, was well approximated by the theoretically derived curve [26], confirming that a matrix of independent SNPs ascertained in GWAS qualifies as a CS sensing matrix.

The noiseless case corresponds to a trait with a perfect narrow-sense heritability (*h*^2^ = 1). Although there are some phenotypes that approach this ideal situation, it is important to consider the more typical situation of *h*^2^ < 1. Figure 1B shows how the *NE* varied in the presence of a noise level corresponding to *h*^2^ = 0.5 (which is roughly the SNP-based heritability of height [7,30]). We can see that the transition boundary was smoothed and effectively shifted downward.

In the noisy case, the transition boundary was less dependent on *δ* than in the noiseless case. Note that in Figure 1A-B the noise variance is fixed by *h*^2^, but *ρ* and *δ* are both functions of the sample size. Fixing *ρ* and traversing the phase plane horizontally can be interpreted as using a sample of size *n* to study a particular phenotype with *s* nonzeros, changing the number of genotyped markers in successive assays; Figure 1B shows that in the noisy case an order-of-magnitude change in *p* had a negligible impact on the quality of selection.

Given this insensitivity to *δ*, it is instructive to increase the resolution with which the phase transition can be studied by fixing *δ* and then comparing the *h*^2^ = 1 and *h*^2^ = 0.5 cases. Figure 1C shows that the *NE* approached its asymptote beyond the theoretical phase transition in both cases. Moreover, the asymptote appeared to be greater than zero in the noiseless case. This behavior may suggest that the noise-dependent *λ*_min_ prescribed by CS theory is suboptimal when noise is in fact absent; although the closeness of the theoretical and empirical phase boundaries implies that the deviation from optimality is mild. The transition was not altered in the noiseless case when *λ*_min_ was estimated using cross-validation, although there was some improvement in the noisy case. A 10-fold cross-validation increased the computational time by 10 to 100-fold. The similar quality of selection achieved by the theoretical *λ*_min_ and the use of cross-validation supports the theoretical estimate.

In the noiseless case, when using a criterion of *NE* < 0.5, the phase transition to vanishing *NE* began at *ρ* ≈ 0.4. In the noisy case of *h*^2^ = 0.5, the phase transition began at *ρ* ≈ 0.03 (*n* ≈ 30*s*). As expected, the sample size for a given number of nonzero coefficients must be larger in the presence of noise.

### Measures of selection

We next examined whether nonzeros were being correctly selected despite a nonzero *NE* by considering additional measures of selection:

1. The false positive rate (*FPR*), the fraction of true zero-valued coefficients that are falsely identified as nonzero.

2. The positive predictive value (*PPV*), the number of correctly selected true nonzeros divided by the total number of nonzeros returned by the selection algorithm. 1 − *PPV* equals the false discovery rate (*FDR*).

3. The median of the *P*-values obtained when regressing the phenotype on each of the *L*_1_-selected markers in turn (*μ*_*P* − value_). Each such *P*-value is the standard two-tailed probability from the *t* test of the null hypothesis that a univariate regression coefficient is equal to zero. The previous measures of recovery—*NE*, *FPR, PPV*—cannot be computed in realistic applications because they depend on the unknown **x**, and thus it is of interest to examine whether an observable quantity such as *μ*_*P* − value_ also undergoes a phase transition at the same critical sample size.

We hypothesized that a measure of the *P*-value distribution of the putative nonzero set may reflect the phase transition since the distribution of *P*-values of normally distributed random variables is uniform and is the basis of false discovery approaches for the multiple comparisons problem [43].

**Figure 2:**
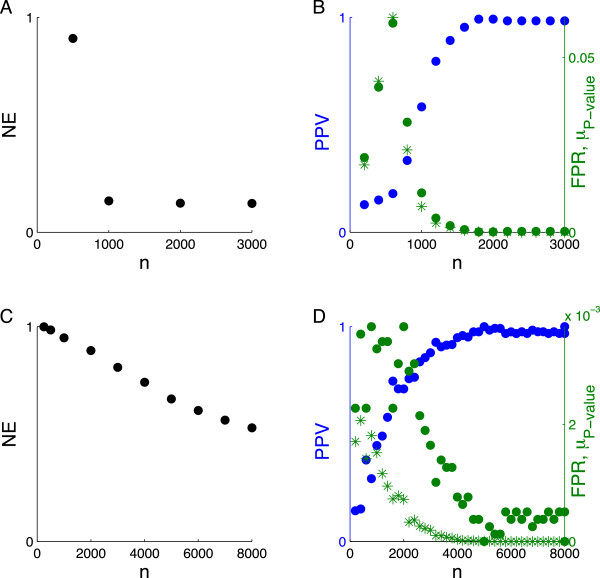
**Measures of selection as a function of sample size for the measurement matrix of random genomic SNPs.** Fixing *s* = 125 and *p* = 8,027, we measured the selection of true nonzero coefficients according to four metrics for *h*^2^ = 1 **(A-B)** and *h*^2^ = 0.5 **(C-D)**. Shown in **(A-C)** is the normalized error of the coefficients (*NE*). Shown in **(B-D)** are the positive predictive value (*PPV*, blue dots), false positive rate (*FPR*, green dots), and median *P* -value (*μ*_*P* − value_, green asterisks). The point *n* = 1, 000 corresponds to (*ρ* = 0.125, *δ* = 0.125) and *n* = 5, 000 to (*ρ* = 0.025, *δ* = 0.625) noted in Figure 1**A** and **B** respectively.

We now turn to the behavior of these performance metrics as a function of sample size. In the noiseless case (Figure 2A-B), the *NE* showed a phase transition at *n ≈* 1,000, but the *PPV*, *FPR* and *μ*_*P* − value_ converged around *n =* 1,500. Since we fixed *s* to be 125, the location of the transition boundary with respect to the *NE* at the point (*ρ* = 0.125, *δ* = 0.125) was consistent with Figure 1A. Also shown is the point (*ρ* = 0.08, *δ* = 0.19), where the *PPV*, *FPR*, and *μ*_*P* − value_ converged. As the noise was increased (Figure 2C), the *NE* declined less sharply with increasing *n*, as expected from Figure 1. In contrast and shown in Figure 2D, the other measures (particularly the *PPV* and *μ*_*P* − value_) neared their asymptotic values even in the presence of noise. The transitions of *FPR, PPV*, and *μ*_*P* − value_ from poor to good performance were not smoothed by noise to the same extent as the transition of the *NE*.

The greater robustness of the *FPR*, *PPV* and *μ*_*P* − value_ against residual variance relative to the *NE* shows that accurate *selection* of nonzeros can occur well before the precise *fitting* of their coefficient magnitudes. The fact that the observable quantity *μ*_*P* − value_ exhibits this robustness is particularly important; a steep decline in *μ*_*P* − value_ across subsamples of increasing size drawn from a given dataset demonstrates a transition to good recovery and implies that the full dataset has sufficient power for accurate identification. This is an empirical finding that deserves further investigation.

For *h*^2^ = 0.5 and across all measures of performance other than the *NE*, the transition appeared to be around *n* = 5,000. Given *s* = 125 and *p* = 8,027, this corresponds to (*ρ* = 0.025, *δ* = 0.625), which is circled in Figure 1B. This estimate of the critical *ρ* is consistent with our previous estimate when *δ* was fixed at 0.5, supporting the weak dependence on *p*.

### Quality of selection in the presence of LD

We have shown that randomly sampled SNPs from a GWAS of Europeans have the properties of a compressed sensor. This was expected, given that randomly sampled markers will be mostly uncorrelated and therefore closely estimate an isotropic matrix.

**Figure 3:**
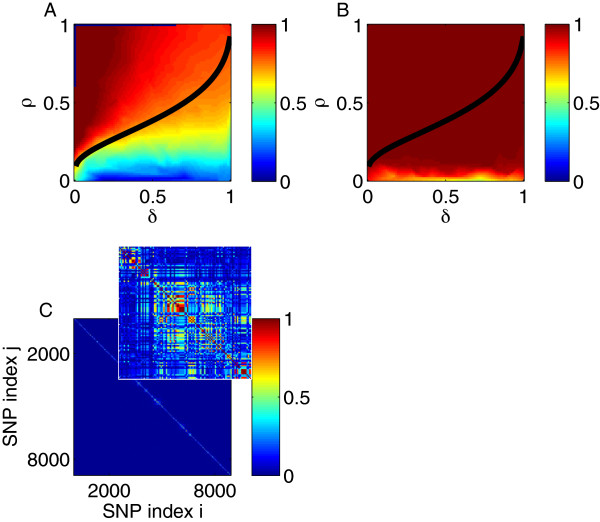
**Analysis of chromosome 22. (A)** The *ρ* − *δ* plane for *h*^2^ = 1. *p* was set to 8,915. Superimposed is the expected phase boundary when there is neither noise nor LD [26]. **(B)** The same as panel **(A)**, except for *h*^2^ = 0.5. **(C)** The matrix of correlations (positive roots of the *r*^2^ LD measure) between genotyped SNPs on chromosome 22. Inset is a 100 × 100 sample along the diagonal.

We next consider a genotype matrix characterized by LD. To do this, while still being able to evaluate recovery at all points of the *ρ* − *δ* plane, we considered all genotyped markers only on chromosome 22. Almost all of these markers were in LD with a few other markers, and the markers within each correlated group tended to be spatially contiguous (Figure 3C). As shown in Figure 3A and B, the phase transition boundary with respect to *NE* was shifted to lower values of *ρ* and was less sensitive to *δ* as in Figure 1B.

**Figure 4:**
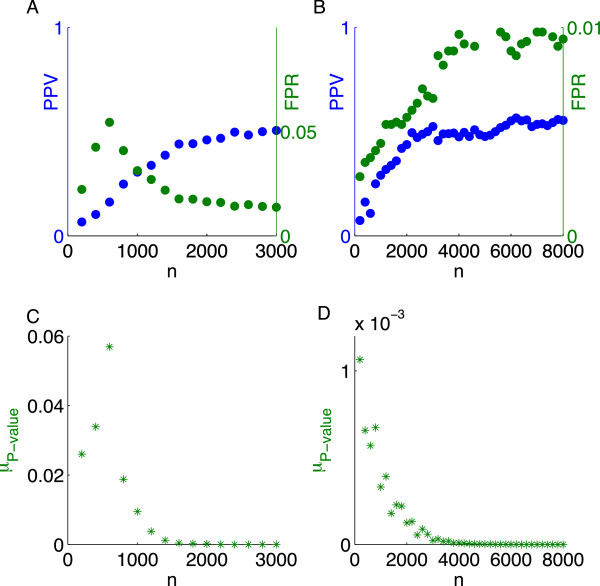
**Measures of selection as a function of sample size for chromosome 22 (*s*** **=** **125 and *p*** **=** **8,** **915).** The PPV (blue) and FPR (green) for *h*^2^ = 1 **(A)** and *h*^2^ = 0.5 **(B)**. *μ*_*P* − *value*_ for *h*^2^ = 1 **(C)** and *h*^2^ = 0.5 **(D)**.

Although the phase transition from large to small *NE* appeared to be affected adversely by LD (at least in the noiseless case as shown in Figure 3A), the selection measures were less affected, as seen by comparing Figure 4 calculated using the intact chromosome 22 with Figure 2 using markers drawn at random from across the genome. Regardless of LD, the transition from poor to good values of *μ*_*P* − value_ occurred at nearly the same sample size (about 30 times the number of nonzeros for *h*^2^ = 0.5). The *PPV* and *FPR* saturated at worse asymptotic values in the noiseless case. In the noisy case, the *PPV* was also lower; perhaps surprisingly, the *FPR* actually increased with sample size.

The relatively poor performance of the *PPV* and *FPR* in the case of LD is somewhat misleading. For example, an “off-by-one” (nearby) nonzero called by *L*_1_-penalized regression will not count toward the numerator of the *PPV*, even if it is in extremely strong LD with a true nonzero. At the same time, such a near miss does count toward the numerator of the *FPR* This standard of recovery quality seems overly stringent when we recall that picking out the causal variant from a GWAS “hit” region containing multiple marker SNPs in LD continues to be a challenge for the standard MR approach [44,45].

**Figure 5:**
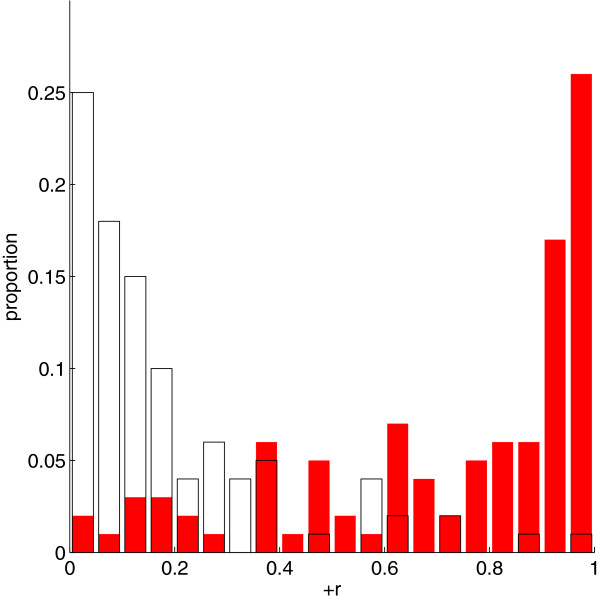
**Distribution of maximum correlations between false positives and true nonzeros after the presumptive *μ***_***P*** **−** ***value***_**phase transition for chromosome 22.** Histogram of the maximum correlation (maximum of the positive roots of the *r*^2^ LD measure) between a false positive and true nonzero for chromosome 22, given *s* = 125, *n* = 5,000, and *h*^2^ = 0.5 (red). Also shown is one realization from the null distribution, generated by drawing an equal number of “false positives” at random from chromosome 22 (white).

**Figure 6:**
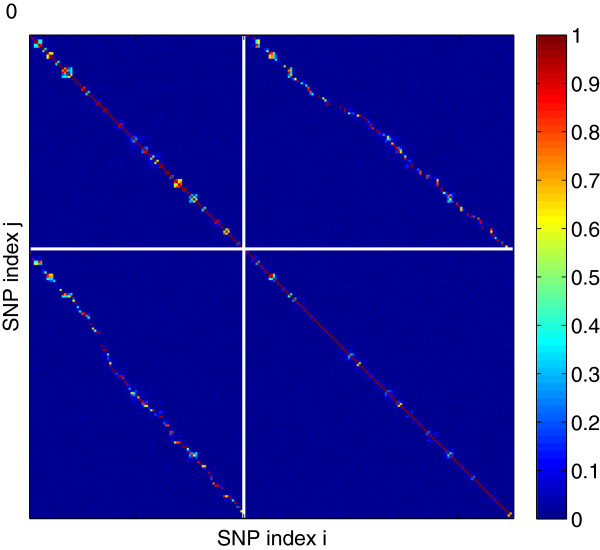
**The matrix of correlations (positive roots of the *r***^**2 **^**LD measure) among false positives and true nonzeros after the presumptive *μ***_***P*** **−** ***value***_**phase transition for chromosome 22 (*s*** **=** **125, *n*** **=** **5,** **000, and *h***^**2**^ **=** **0.5).** SNP indices begin at the top left corner. The upper-left quadrant contains the correlations among false positives and the lower-right quadrant contains the correlations among the true nonzeros. Each element in the upper-right (lower-left) quadrant represents a correlation between a false positive and a true nonzero. Within both the false positive and the true nonzero sets, the markers are arranged in order of chromosomal map position.

We examined whether the false positives called by the *L*_1_-penalized algorithm were indeed more likely to be in strong LD with the true nonzeros by computing the correlations between false positives and true nonzeros for *n* = 5,000 and *h*^2^ = 0.5. Figure 5 shows the histogram of the maximum correlation between each false positive and any of the true nonzeros. We compared this histogram to a realization from the null distribution, generated by drawing markers at random from chromosome 22 and finding each marker’s largest correlation with any of the true nonzeros. The observed histogram featured many more large correlations than the realization from the null distribution, implying that the false positives showed a significant tendency to be in LD with true nonzeros.Figure 6 provides a visualization of the correlations among the false positives and true nonzeros. High correlations between false positives (upper left panel) and between true nonzeros (lower right panel) lie near the main diagonal of self-correlations indicating spatial proximity of correlated SNPs as expected from the LD structure shown in Figure 3C. There are also high correlations between false positives and true nonzeros (upper right and lower left panels). These high correlations are also mostly confined to spatially proximate SNPs demonstrating a marked tendency for called false positives to occur close to one of the true nonzeros.

### Sensitivity to the distributions of coefficient magnitudes and MAF

**Figure 7:**
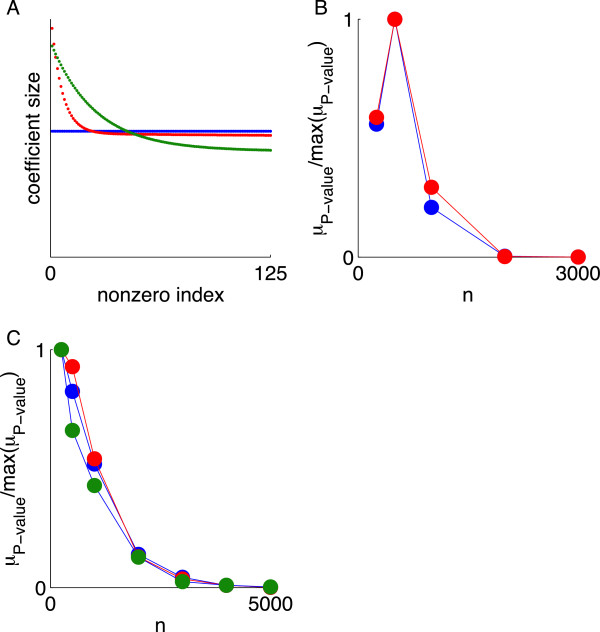
**Insensitivity of the selection phase boundary to the distribution of coefficient magnitudes (ensemble). (A)***s* = 125 coefficient magnitudes (“effect sizes”) ordered from large to small for the Uniform (blue), Hyperexponential 1 (red), and Hyperexponential 2 (green) ensembles. **(B)** Chromosome 22 analysis using *μ*_*P* − value_ to measure selection (normalized by the maximum value) as a function of sample size for *h*^2^ = 1 for the Uniform (blue) and Hyperexponential 1 (red) ensembles. **(C)** As in panel **(B)** except for *h*^2^ = 0.5. Also shown is recovery for the Hyperexponential 2 ensemble (green).

The appropriate prior on the distribution of coefficient magnitudes is often discussed [19]. However, CS theory shows that the phase boundary for complete *selection* is relatively insensitive to this distribution. To test this prediction, we looked for evidence of performance degradation upon replacing the discrete distribution of nonzero coefficients used thus far with a hyperexponential distribution (a mixture of exponential distributions with different decay constants) (these are defined in Data Description and shown in Figure 7A). The hyperexponential distribution is a means of implementing an arguably more realistic ensemble of a few large coefficients followed by a tail of weaker values [38]. Figure 7B-C shows that, as predicted by theoretical CS results, for fixed *h*^2^ and chromosome 22, the normalized *μ*_*P* − value_ converged to zero at the same sample size regardless of the ensemble.

**Figure 8:**
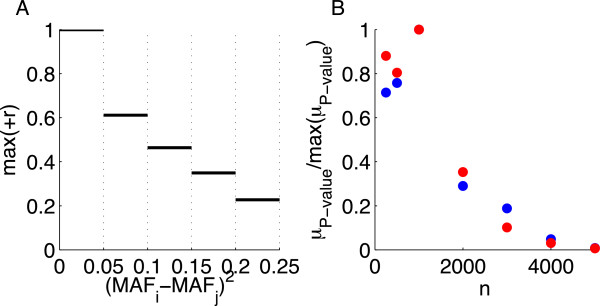
**Insensitivity of the selection phase boundary to minor allele frequency (MAF) for chromosome 22. (A)** The maximum positive root of the *r*^2^ LD measure (+r) as a function of squared MAF difference. The maxima are estimated over bin lengths of 0.05 for SNPs in chromosome 22. **(B)** The median *P* -value (*μ*_*P* − value_) normalized by the maximum value as a function of sample size for *s* = 125 from {−1,  1} and *h*^2^ = 0.5 for nonzero coefficients sampled from low (blue) or high (red) MAF SNPs on chromosome 22.

In the previous simulations, we drew the nonzeros at random from all genotyped markers, thus guaranteeing that the MAF spectra of the nonzeros and the entire genotyping chip would tend to coincide. Here, we also tested whether the MAF spectrum of nonzeros affects the selection phase boundary. It is known that two SNPs can be in strong LD only if they have similar MAFs [46,47]. We confirmed this by taking all pairs of markers on chromosome 22 and plotting the maximum positive root of the LD measure as a function of squared MAF difference (Figure 8A). Therefore, in order to isolate any effect of the MAF distribution among nonzeros not mediated by LD, we constructed a synthetic measurement matrix **A** with independent columns and the same MAF spectrum as chromosome 22. We then compared recovery when the nonzero coefficients were sampled from SNPs with MAF between 0.0045 and 0.015, or when they were sampled above MAF of 0.49. For this we used nonzeros from {−1,  1}. Figure 8B shows no difference in recovery between the conditions for *h*^2^ = 0.5. This suggests that MAF alone is not a determinant of the phase transition. Homogeneity in MAF among nonzeros may enrich correlations as noted above. Such correlations would be expected to reduce the effective *s* and thus affect the phase boundary.

### Selection of SNPs associated with height

Motivated by the results above, we examined whether the full sample size of 12,454 subjects was sufficient to achieve the phase transition from poor to good recovery of SNPs associated with a real phenotype (height). We considered the selection measures *μ*_*P* − value_ and adjusted the positive predictive value (PPV*); the latter extended true-positive status to any selected SNP within 500 kb of a SNP identified as a likely marker of a height-affecting variant in the GIANT Consortium’s analysis of ~ 180,000 unrelated individuals [39]. This extension is consistent with the rule of thumb designating a 1-Mb region as a “locus” for purposes of counting the number of GWAS “hits” [48]. The relative insensitivity of *μ*_*P* − value_ to LD suggests that PPV* rewards the identification of both true nonzeros and markers tagging nonzeros; we therefore substituted PPV* for *PPV* in an attempt to align the phase dynamics of our precision measure with those of *μ*_*P* − value_. Whether a selected marker fell within 500 kb of a GIANT-identified marker was determined by consulting the Broad Institute’s SNAP database [40].

**Figure 9:**
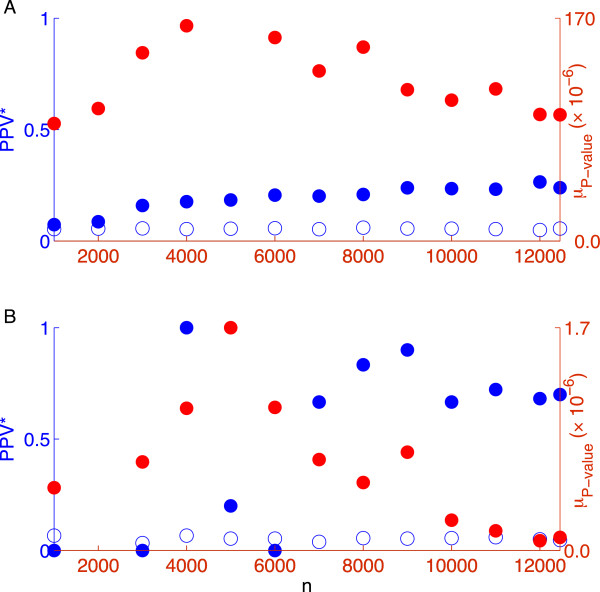
**Selection measures as a function of sample size in an analysis of real height data. (A)** The adjusted positive predictive value (PPV*, blue solid dots) and median *P* -value (*μ*_*P* − value_, red) as a function of sample size using *λ* based on *h*^2^ = 0.5. Also shown is PPV* when the same number of SNPs are randomly selected rather than returned by the *L*_1_ algorithm (blue unfilled dots). **(B)** As in **(A)** but setting *λ* to a value appropriate for *h*^2^ = 0.01.

Figure 9A shows that *μ*_*P* − value_ failed to approach zero, suggesting that that *n* = 12, 454 is not large enough to see a phase transition to the regime of good recovery. Given our empirical finding that *ρ* ≈ 0.03 is required for *h*^2^ ≈ 0.5, this suggests that height is affected by at least 400 causal variants, a result consistent with the observation that the ~250 known height-associated SNPs account for only a small proportion of this trait’s additive genetic variance [48]. However, the null PPV* derived from randomly chosen SNPs was smaller than the observed PPV* (Figure 9A); this was consistent with the detection of some true signal. In other words, although no phase transition was evident, the recovery measure did improve with increased sample size.

The penalization parameter *λ* was set using CS theory to minimize NE error based on the expected noise-level from reported narrow sense heritability for height [7,30]. If *λ* is set too low, then more false positives are expected; if *λ* is set too high, then true nonzeros will be missed. According to CS theory, an *L*_1_-penalized method can still select some of the largest coefficients from a non-uniform distribution of coefficient magnitudes even if complete recovery is out of reach [49]. We investigated whether it was possible to achieve a phase transition to low *μ*_*P* − value_ and high PPV*, at the cost of recovering only a small fraction of all true nonzeros, by increasing the penalty parameter *λ*. More specifically, we set *λ* to a higher value consistent with *h*^2^ = 0.01 rather than 0.5. In this case, the *L*_1_ algorithm returned 20 putative nonzeros rather than the original 403, and both *μ*_*P* − value_ and PPV* exhibited better performance (Figure 9B). Compared to the less stringent λ, PPV* as a function of *n* was less smooth, but appeared to stabilize to a high recovery value after ∼ 7000 subjects. Evidently, if the sample size does not suffice to capture the full heritability, setting the penalty parameter to a value appropriate for a lower heritability can lead to a smaller set of selected markers characterized by good precision.

**Figure 10:**
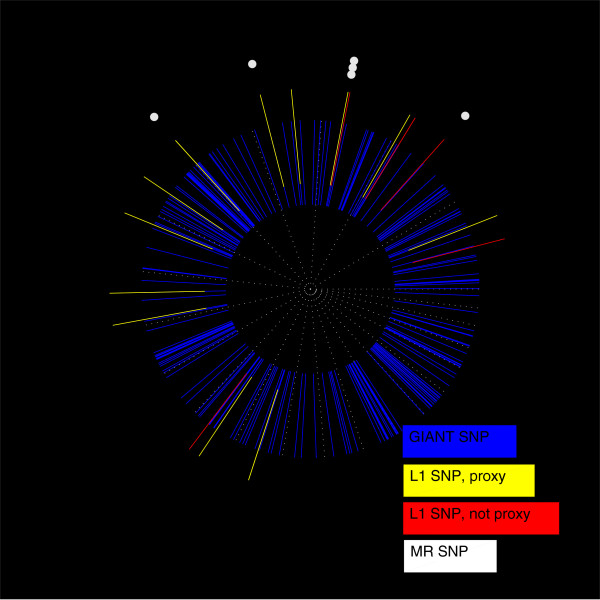
**Map of SNPs associated with height, as identified by the GIANT Consortium meta-analysis, *L***_**1 **_**-penalized regression, and standard GWAS.** Base-pair distance is given by angle, and chromosome endpoints are demarcated by dotted lines. Starting from 3 o’clock and going counterclockwise, the map sweeps through the chromosomes in numerical order. As a scale reference, the first sector represents chromosome 1 and is ∼ 250 million base-pairs. The blue segments correspond to a 1 Mb window surrounding the height-associated SNPs discovered by GIANT. Note that some of these may overlap. The yellow segments represent *L*_1_ -selected SNPs that fell within 500 kb of a (blue) GIANT-identified nonzero; these met our criterion for being declared true positives. The red segments represent *L*_1_ -selected SNPs that did not fall within 500 kb of a GIANT-identified nonzero. Note that some yellow and red segments overlap given this figure’s resolution. There are in total 20 yellow/red segments, representing *L*_1_ -selected SNPs found using all 12,454 subjects. The white dots represent the locations of SNPs selected by MR at a *P* -value threshold of 10^− 8^.

Figure 10 illustrates the physical distances between the markers selected in our strict-*λ* (assuming *h*^2^ = 0.01) analysis and the markers identified by the GIANT Consortium. Of the 20 *L*_1_- selected markers, 14 were within 500-kb of a GIANT-identified marker. However, the *L*_1_-selected markers defined to be false positives were still relatively close to GIANT-identified markers. This may indicate that the 500-kb criterion for declaring a true positive was too stringent; if so, then our stated PPV* of 0.7 can be regarded as a lower bound. As an informal comparison, Figure 10 also displays the results of a more standard MR-type GWAS analysis. For a *P*-value of 10^− 8^ and all 12,454 subjects, MR returned six SNPs, five of which were GIANT-identified markers, and four were exact matches with SNPs selected by our *L*_1_ algorithm (Figure 10). With a *P* -value cutoff of 5 × 10^− 8^ and all subjects, MR returned 13 markers, 10 of which were GIANT-identified, and 7 of which were identical to the *L*_1_ -selected markers.

**Figure 11:**
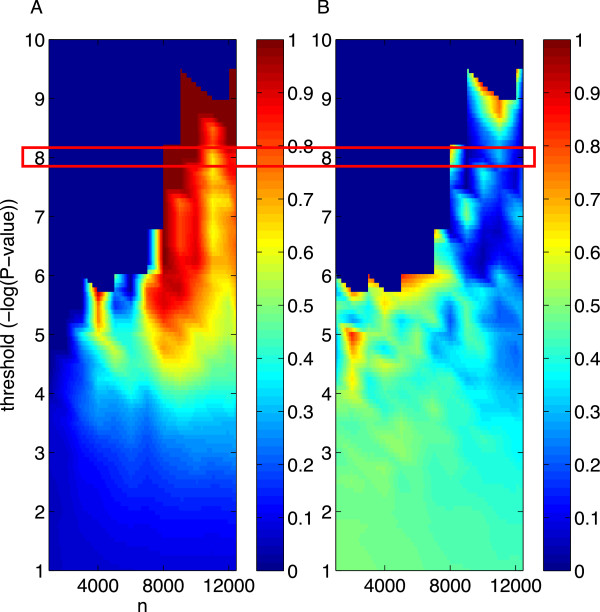
**Measures of recovery using marginal regression (standard GWAS) as a function of sample size.** All SNPs surviving the chosen − log_10_*P* − value threshold were selected. The recovery measures, computed over the selected SNPs, were **(A)** the adjusted positive predictive value (PPV*) and **(B)** the median *P* -value divided by the *P* -value cutoff. Highlighted in red is the cutoff we used for MR in Figure 10.

The presence of a phase transition is not necessarily restricted to *L*_1_ algorithms, but rather may represent a deeper phenomenon in signal recovery. Other methods may show a similar phase transition—although CS theory suggests that, among convex optimization methods, those within the *L*_1_ class are closest to the optimal combinatorial *L*_0_ search. We conducted additional analyses to test whether a phase transition at a critical sample size could also be observed when our height data were analyzed using the MR approach commonly used in GWAS. In these simulations we varied the *P* -value threshold for genome-wide significance. As measures of selection are potentially subject to a phase transition, we examined the PPV* and the adjusted median *P* -value ($\mu_{P-\mathrm{value}}^{*}$). The latter measure was defined to be the median *P* -value among those SNPs surviving the *P* -value cutoff, divided by the cutoff itself; the normalization was necessary to remove the dependence on the choice of cutoff. As shown in Figure 11, the *P* -value threshold 10^− 8^ yielded very few selected SNPs, and in fact, none were returned at sample sizes smaller than approximately 8,000. However, $\mu_{P-\mathrm{value}}^{*}$ was mostly close to zero in the region of Figure 11B corresponding to *n* > 8, 000 and *P* − value < 10^− 6^, suggesting that true nonzeros were being selected. This is confirmed by the fact that the PPV* typically exceeded 0.6 in this same region (Figure 11A). For *P* -value thresholds less stringent than 10^− 6^, signs of a phase transition at a critical sample size were still discernible.

A search for a phase transition can be a useful approach to determining the optimal *P* -value threshold in standard GWAS protocols employing MR. In addition to *a priori* assumptions regarding the likely number of true nonzeros and their coefficient magnitudes [38,50] and agreement between studies of different designs [51], GWAS investigators might rely on whether a measure such as $\mu_{P-\mathrm{value}}^{*}$ undergoes a clear phase transition as they take increasingly large subsamples of their data. A majority of markers surviving the most liberal significance threshold bounding the second phase are likely to be true positives.

## Discussion

Our results with real European GWAS data and simulated vectors of regression coefficients demonstrate the accurate selection of those markers with nonzero coefficients, consistent with CS sample size requirements (*n*) for a given sparsity (*s*) and total number of predictors (*p*). We found that the matrix of standardized genotypes exhibits the theoretical phase transition between poor and complete selection of nonzeros (Proposition 1). We also found, as for Gaussian random matrices in earlier studies, that the phase transition depends on the scaling ratios *ρ* = *s*/*n* and *δ* = *n*/*p*[42].

We obtained results regarding the effect of noise (i.e., *h*^2^ < 1) that are consistent with earlier empirical studies of random matrices and recently proven theorems [22,24,28]. Generally speaking, we show that the critical sample size is determined mainly by the ratio of *s* to *n* and only weakly sensitive to *p*, particularly as noise increases. For example, if *h*^2^ = 0.5, which is roughly the narrow-sense heritability of height and a number of other quantitative traits [7,30,37], we find that *ρ* should be less than approximately 0.03 for recovery irrespective of *δ*. There is no hope of recovering the complete vector of coefficients **x** above this threshold (i.e., smaller sample sizes). For example, if we have prior knowledge that *s* = 1, 200, then this means that the sample size should be no less than 40,000 subjects. We find empirically that for *h*^2^ ∼ 0.5, *n* ∼ 30*s* is sufficient for selection of the nonzeros.

In real problems we cannot rely on measures of model recovery based on the unknown **x**. Hence, we introduced a new measure based on the median *P* -value of the *L*_1_ -selected nonzeros, *μ*_*P* − value_. We found that *μ*_*P* − value_ provides a robust means of detecting the boundary between poor and good recovery. Proposition 2 shows that the recovery error *NE* in the favorable phase scales with *ρ* and noise; however, we observed that the recovery measures *FPR*, *PPV* and *μ*_*P* − value_ approached zero faster than the *NE*, confirming that accurate identification of nonzeros can occur well before precise estimation of their magnitudes.

An *L*_1_ -penalized regression algorithm is equivalent to linear regression with a Laplace prior distribution of coefficients, and in theory a Bayesian method invoking a prior distribution better matching the unknown true distribution of nonzero coefficients should outperform the lasso in effect estimation. However, it is by no means clear that the performance of *L*_1_ penalization with respect to selection can be bettered. For example, the lasso and BayesB display rather similar performance properties [17]. However, both methods clearly outperformed ridge regression (a non- *L*_1_ method), which exhibited no phase transition away from poor performance. Furthermore, it is usually accepted by GWAS researchers that knowledge of the markers with nonzero coefficients may be quite valuable, even if the actual magnitudes of the coefficients are not well determined. Combining the advantages of different approaches by applying one of them to the *L*_1_ -selected markers is a possibility.

Perhaps contrary to intuition, but consistent with theoretical results for CS [25,42], we found that the phase transition to good recovery (at least as measured by *μ*_*P* − value_) was insensitive to the distribution of coefficient magnitudes. It is well known in CS that *L*_1_ -penalized regression is nearly minimax optimal (minimizes the error of the worst case), and that the phase transition is robust to the distribution of coefficient magnitudes. In some cases a good prior may reduce the mean-square error and shift the location of the phase transition [52]. However, simulations supporting this notion, were performed with a much higher signal-to-noise ratio (SNR) than hypothesized for realistic GWAS problems. The performance increase was attenuated as the SNR was decreased to levels still higher than usual in GWAS (10 dB or *h*^2^ > 0.9 where SNR on the dB scale is given by $10\cdot \log_{10}\left(\frac{\sigma_{A}^{2}}{\sigma_{E}^{2}}\right)$). These algorithms are currently being explored in lower-SNR regimes. We observed that cross-validation did slightly affect the phase transition boundary in the noisy case; nevertheless the theoretical penalization parameter proved to be a good rule of thumb for initial screening. Calculating the theoretical penalty depends on knowledge of *h*^2^, which may be estimated using the genomic-relatedness method [7,30-32].

Genomic selection methods have been criticized by researchers who doubt that the number of nonzeros (*s*) will typically be smaller than a practically attainable sample size (*n*) [19]. The application of CS theory circumvents this problem because it allows the optimization method to self-determine whether or not the nonzero markers are sufficiently sparse compared to the sample size. No prior assumptions are required. Furthermore, there is evidence that a number of traits satisfy the sparsity assumption in humans, at least with respect to common variants contributing to heritability [9-11].

CS theory does not provide performance guarantees in the presence of arbitrary correlations (LD) among predictor variables: it must be verified empirically, as we have done. In agreement with previous results [17], we find that the phase transition, as measured by NE, is strongly affected by LD. However, according to our simulations using all genotyped SNPs on chromosome 22, *L*_1_ -penalized regression does select SNPs in close proximity to true nonzeros. The difficulty of fine-mapping an association signal to the actual causal variant is a limitation shared by all statistical gene-mapping approaches—including marginal regression as implemented in standard GWAS—and thus should not be interpreted as a drawback of *L*_1_ methods.

We found that a sample size of 12,464 was not sufficient to achieve full recovery of the nonzeros with respect to height. However, the penalization parameter *λ* is set by CS theory so as to minimize the *NE* based on the expected noise-level. In some situations it might be desirable to tolerate a relatively large *NE* in order to achieve precise, but incomplete recovery (few false positives, many false negatives). By setting *λ* to a strict value appropriate for a low-heritability trait (in effect, looking for a subset of markers that account for only a fraction of the total heritability, with consequently higher noise), we found that a phase transition to good recovery can be achieved with smaller sample sizes, at the cost of selecting a smaller number of markers and hence suffering many false negatives.

One interesting feature of the recovery measure based on the median *P* -value (*μ*_*P* − value_) is that it seemed to rise as the sample size was increased in the region of poor recovery and then fall after the sample size crossed the CS-determined phase transition boundary. This rise and then fall was very dramatic in our simulations (Figures 2 and 4) and also appeared in our analysis of height (Figure 9). This behavior may be a consequence of the fact that as the sample size is increased, *λ* in the algorithm is decreased (see Methods). Hence, in the region of poor recovery, the relaxation of the penalty with increasing sample size may permit the selection of more SNPs and hence the inflation of the *FPR* and *μ*_*P* − value_. However, once the phase transition to good performance begins, the recovery measures begin their characteristic sharp decrease. This non-monotone behavior accentuates the transition boundary and can be exploited to aid its detection.

In summary, compressed sensing utilizes properties of high-dimensional systems that are surprising from the perspective of classical statistics. The regression problem faced by GWAS and GS is well-suited to such an approach, and we have shown that the matrix of SNP genotypes formed from European GWAS data is in fact a well-conditioned sensing matrix. Consequently, we have inferred the sample sizes required to achieve accurate model recovery and demonstrated a method for determining whether the minimal sample size has in fact been obtained.

## Methods

### ***L***_1_-penalized regression algorithm

*L*_1_-penalized regression (e.g., lasso) minimizes the objective function

(2) $${∥\;\hat{y}-y\;∥}_{L2}^{2}+\;{∥\hat{x}\;∥}_{L_{1}}$$

where $\hat{y}$ is the estimated breeding value given by $A\hat{x}$. The setting of the penalization parameter *λ* is described below.

The algorithm was performed using pathwise coordinate optimization and the soft-threshold rule [53]. Regression coefficients were sequentially updated with

(3) $$\begin{array}{ll} {\hat{x}}_{j} & \left(\lambda \right)\leftarrow S\left({\hat{x}}_{j}\left(\lambda \right)+\frac{1}{n}\sum_{i=1}^{n}A_{\mathrm{ij}}\left(y_{i}-{\hat{y}}_{i}\right),\lambda \right)\mathrm{for}\;j\\ & =1,2,\ldots ,p \end{array}$$

where

(4) $$\begin{array}{l} S\left(z,\lambda \right)\equiv \mathrm{sign}\left(z\right)(|z|-\lambda ){}_{+}\\ =\left\{\begin{array}{cc} z-\lambda , & \mathrm{if}\;z>0\;\mathrm{and}\;\lambda <|z|,\\ z+\lambda , & \mathrm{if}\;z<0\;\mathrm{and}\;\lambda <|z|,\\ 0, & \mathrm{if}\;\lambda \geq |z| \end{array}\right) \end{array}$$

We assumed convergence if the fractional change in the objective function given by Equation 2 was less than 10^− 4^. In addition, we performed lasso with a warm start [54], using a logarithmic descent of 100 steps in *λ* with *λ*_max_  =  ($\frac{1}{n}$) ∥ **Ay** ∥_*L* ∞_. For *λ*_min_ we used $\left(\sigma_{E}^{*}/n\right)\;{∥\;\mathrm{Ae}\;∥}_{L\infty }$, where $\sigma_{E}^{*}=\sqrt{\sigma_{E}^{2}+\frac{1}{n}}$[22]. To estimate ∥**A** ' **e**∥_*L*∞_ we created 1,000 sample vectors of **e**, each constructed with *n* i.i.d. normal elements with mean zero and variance one, and took the median across samples of ∥**A** ' **e**∥_*L*∞_. Estimates of $\left(\sigma_{A}^{2},\sigma_{E}^{2}\right)$ with respect to the variants assayed in a given study can be obtained using the genomic-relatedness method [7,30-32]. The algorithm can also accommodate any other covariates.

### Computations

Simulations and analyses were performed using MATLAB 2013 (The MathWorks Inc., Natick, Massachusetts) and PLINK 2 [35,36]. The *L*_1_ -optimization algorithm was written in MATLAB and also a feature of PLINK 2. *P* -values were estimated using MATLAB’s *regstats* function and PLINK 2. Color-coded phase plane figures were generated by sampling the *ρ* − *δ* plane and interpolating between points using MATLAB’s *scatteredInterpolant* function. GWAS data were obtained from dbGaP as described in Data Description. Analysis scripts are available from the *GigaScience* GigaDB repository and maintained on GitHub [55,56].

### Statistics

The normalized coefficient error (*NE*) is

(5) $$\frac{{∥\;x\;-\;\hat{x}∥}_{L2}}{{∥\;x\;∥}_{L2}}$$

The false positive rate (*FPR*) is the fraction of true zero-valued coefficients that are falsely identified as nonzero. The positive predictive value (*PPV*) is the number of correctly selected true nonzeros divided by the total number of nonzeros returned by the selection algorithm. 1 − *PPV* equals the false discovery rate (*FDR*). The adjusted positive predictive value (PPV*) is similar to the standard *PPV*, except that any selected nonzero coefficient falling within 500 kb of a GIANT-identified marker is counted as a true positive [39].

The median of the *P* -values for the set of putative nonzeros (*μ*_*P* − *value*_) is obtained by: 1) regressing the phenotype on each of the *L*_1_ -selected markers in turn, 2) estimating each *P* -value as the standard two-tailed probability from the *t* test of the null hypothesis that a univariate regression coefficient is equal to zero, and 3) taking the median over the independent tests. This procedure is independent of the selection algorithm and calculated after the *L*_1_ -penalized algorithm has converged. The adjusted median *P* -value ($\mu_{P-\mathrm{value}}^{*}$) is the median of the MR *P* -values falling below the significance threshold divided by the threshold itself.

The LD measure (*r*^2^) is the squared estimate of the Pearson’s product–moment correlation between the standardized zero-mean, unit-variance SNPs.

Analysis codes are archived in the *GigaScience* GigaDB repository and maintained on GitHub [55,56].

## Availability of supporting data

As noted above, the data sets supporting the results of this article are available through dbGaP accession numbers [ARIC:phs000090] and [GENEVA:phs000091], http://www.ncbi.nlm.nih.gov/gap[34]. Mock data sets supporting the results of this article are available in the GigaDB repository, doi:10.5524/100094 and http://gigadb.org/dataset/view/id/100094/[55].

## Abbreviations

ARIC: Atherosclerosis risk in community; CS: Compressed sensing; FDR: False discovery rate; FPR: False positive rate; GENEVA: Gene environment association studies; GIANT: Genetic investigation of anthropometric traits; GS: Genomic selection; GWAS: Genome-wide association study; LD: Linkage disequilibrium; LE: Linkage equilibrium; MAF: Minor allele frequency; MR: Marginal regression; NE: Normalized error; OLS: Ordinary least squares; PPV: Positive predictive value; SNP: Single-nucleotide polymorphism.

## Competing interests

The authors declare that they have no competing interests.

## Authors’ contributions

SV performed the numerical experiments and analyzed the data. SV, JJL, SDHH, and Chow contributed to the conception of the study, drafted the article, and endorsed the final version for submission. Chang ported the MATLAB *L*_1_ -penalized regression codes to PLINK 2 for use in the height analysis. All authors read and approved the final manuscript.
